# Efficacy and safety of artemether–lumefantrine for treatment of uncomplicated *Plasmodium falciparum* malaria in Ethiopia: a systematic review and meta-analysis

**DOI:** 10.1186/s12936-021-03745-8

**Published:** 2021-05-06

**Authors:** Abdulhakim Abamecha, Daniel Yilma, Wondimagegn Adissu, Delenasaw Yewhalaw, Alemseged Abdissa

**Affiliations:** 1 School of Medical Laboratory Sciences, Institute of Health, Jimma University, Jimma, Ethiopia; 2 Department of Internal Medicine, Institute of Health, Jimma University, Jimma, Ethiopia; 3 Clinical Trial Unit, Jimma University, Jimma, Ethiopia; 4 Tropical and Infectious Diseases Research Center (TIDRC), Jimma University, Jimma, Ethiopia; 5 Armauer Hansen Research Institute, Addis Ababa, Ethiopia; 6 Department of Biomedical, College of Public Health and Medical Science, Mettu University, Mettu, Ethiopia

**Keywords:** Therapeutic efficacy, Artemether–lumefantrine, *Plasmodium falciparum*, Systematic review, Ethiopia

## Abstract

**Background:**

Regular monitoring of anti-malarial drug efficacy is vital for establishing rational malaria treatment guidelines and ensuring adequate treatment outcomes. This study aimed to synthesize the available evidence on the efficacy of artemether–lumefantrine for the management of uncomplicated falciparum malaria in Ethiopia.

**Methods:**

The Preferred Reporting Items for Systematic Reviews and Meta-Analyses (PRISMA) guidelines were followed. Relevant published studies were searched from the databases (PubMed, Google Scholar and Clinical trial registry) on published artemether–lumefantrine therapeutic efficacy studies conducted in Ethiopia from 2004 to 2020. The retrieved studies were assessed for quality using the modified Newcastle Ottawa Scale for observational studies and modified Jadad scale for interventional studies. Risk of bias was also assessed by using ROBINS-I tool. OpenMeta-Analyst software was used for the statistical analysis. The review protocol is registered in PROSPERO, number CRD42020201859.

**Results:**

Fifteen studies (1523 participants) were included in the final analysis. The overall PCR-uncorrected pooled proportion of treatment success of artemether–lumefantrine therapy for uncomplicated falciparum malaria was 98.4% (95%CI 97.6–99.1). A random-effects model was used because of considerable heterogeneity [χ^2^ = 20.48, *df* (14), *P* = 0.011 and I^2^ = 31.65]. PCR-corrected pooled proportion of treatment success of artemether–lumefantrine therapy was 98.7% (95% CI 97.7–99.6). A random-effects model was used [χ^2^ = 7.37, *df*(6), *P* = 0.287 and I^2^ = 18.69]. Most studies included in the present review achieved a rapid reduction of fevers and parasitaemia between D0 and D3 of assessment. Adverse events were mostly mild and only two cases were reported as serious, but were not directly attributed to the drug.

**Conclusion:**

The present meta-analysis suggests that artemether–lumefantrine therapy is efficacious and safe in treating uncomplicated falciparum malaria in Ethiopia. However, owing to the high risk of bias in the included studies, strong conclusions cannot be drawn. Further high-quality RCTs assessing anti-malarial efficacy and safety should be performed to demonstrates strong evidence of changes in parasite sensitivity to artemether–lumefantrine in Ethiopia.

**Supplementary Information:**

The online version contains supplementary material available at 10.1186/s12936-021-03745-8.

## Background

Malaria is one of the leading health problems in Ethiopia. Approximately 60% of the total populations in Ethiopia live in malaria-endemic area. Due to the unstable nature of malaria transmission in the country, major malaria epidemics had been one of the serious public health emergencies. Sixty percent of malaria infections in Ethiopia are due to *Plasmodium falciparum* and 40% of infections are due to *Plasmodium vivax* [1, 2].

Resistance of *P. falciparum* to the traditional anti-malarial drugs (such as chloroquine, sulfadoxine–pyrimethamine, amodiaquine, and mefloquine) is a growing problem and is thought to have contributed to increased malaria mortality in recent years [3]. Chloroquine resistance has now been documented in all regions except Central America and the Caribbean. There is high‐level resistance to sulfadoxine‐pyrimethamine throughout South East Asia and increasingly in Africa, including Ethiopia, and mefloquine resistance is common in the border areas of Cambodia, Myanmar, and Thailand [3, 4].

To combat the spread of resistance, the World Health Organization (WHO) now recommends that *P. falciparum* malaria should always be treated using a combination of two drugs that act at different biochemical sites within the parasite [3]. If a parasite mutation producing drug resistance arises spontaneously during treatment, the parasite should then be killed by the partner drug, thus reducing or delaying the development of resistance and increasing the useful lifetime of the individual drugs [5, 6]. The current drug combinations all include a short‐acting artemisinin derivative (such as artesunate, artemether, or dihydroartemisinin), partnered with a longer‐acting drug in combinations known as ‘artemisinin‐based combination therapy’ (ACT). In Ethiopia, the use of artemether-lumefantrine (20/120 mg) as the first-line treatment for uncomplicated falciparum malaria has been started in 2004 [7].

The potency of artemisinin and its derivatives such as artemether, dihydroartemisinin, and artesunate is very high against all erythrocytic cycle asexual stages of *P. falciparum* with preference to the young ring stages [8]*,* so much that it reduces the parasite biomass by 100 to 10,000 folds per each asexual blood stage cycle (after 48 h). It also kills young gametocytes, hence playing a role in reducing malaria transmission [9]. The proposed mechanisms by which artemisinins kill the parasites are quite broad and are still being studied, but they generally fall under two categories: (1) Damaging parasite proteins, such as transport proteins through haem-activated endoperoxide activity and (2) Inhibition of proteasome activity (parasite’s cellular repair mechanisms) leading to accumulation of damaged/unfolded proteins and stress-induced death [10–14].

Due to the risk of the emergence and spread of anti-malarial drug resistance, the WHO recommends regular monitoring of anti-malarial drug efficacy at least every 2 years in malaria-endemic countries [15]. In Ethiopia, the Federal ministry of Health (FMOH), in collaboration with its partners, including President’s Malaria Initiative (PMI), research institutions, universities, WHO country office and Global fund, have been conducting regular therapeutic efficacy studies (TESs). The efforts of the FMOH to ensure regular TESs have also been complemented by TESs conducted by independent researchers [16, 17].

A meta-analysis of AL efficacy studies in Ethiopia was also carried out in 2017, but had several limitations including failure to assess risk of bias and missed studies [18, 19]. Hence, this study aimed to synthesize the available evidence, including new studies and studies that were missed in the previous meta-analysis, on the efficacy of AL for the management of uncomplicated falciparum malaria in Ethiopia.

## Methods

### Study protocol registration

The present study adhered to the preferred reporting items for systematic reviews and meta-analyses (PRISMA)guideline [20]. The completed PRISMA checklist is available in Additional file 1. The review protocol was registered in a repository of systematic review protocols prior to starting the research (PROSPERO, protocol number CRD42020201859) [21].

### Searching strategies

The searching strategy was performed using approaches that enhance methodological transparency and improve the reproducibility of the results and evidence synthesis. In this sense, the search strategy was elaborated and implemented prior to study selection, according to the PRISMA checklist as guidance [20]. Additionally, using the Population, Intervention, Comparison, Outcome and Study design (PICOS) strategy [22, 23]. The following major databases were searched: PubMed, Google Scholar, and ClinicalTrials.gov databases. In order to reflect contemporary practice, a search of the literature from the last 16 years (January 2004 to October 2020) was performed. The starting year (i.e., 2004) was purposely chosen because that was the year when Ethiopia adopted use of AL for treating uncomplicated falciparum malaria [24]. The date of the last search was 30th October 2020.

The search terms were developed in line with the Medical Subject Headings (MeSH) thesaurus using a combination of the big ideas (or “key terms”) which derived from the research question. The domains of the search terms were: “efficacy”, “therapeutic efficacy”, “artemether-lumefantrine”, “Coartem”, “*Plasmodium falciparum* malaria”, “falciparum malaria”, “antimalarial drug”, and “Ethiopia”. This study combined terms using the Boolean operator “OR” and “AND” accordingly [25]. Search was limited to studies published in English language until October 2020. Full search strategy for the databases is provided in Additional file 2. Two reviewers (AbAb, and WA) reviewed the search results independently to identify relevant studies. Also, the bibliographic software EndNote X5 citation manager (Thomson Reuters, New York, USA) was used to store, organize and manage all the references and ensure a systematic and comprehensive search.

### Selection criteria

Eligible studies included randomized controlled trials (RCTs), non-randomized single-arm intervention studies (with or without a control group) and prospective cohort studies. This study intended to only include studies with a comparator or control group, but because of the varying quality of papers retrieved, the study methodology deviated from the original methodologic plan and included any study describing patients given a treatment of interest (i.e. AL), which advise a 28-day follow-up to capture cure rate, even if no specific control group was available. All the non-primary literature, retrospective studies, case reports and in vitro experiments were excluded.

A summary of the participants, interventions, comparators and outcomes considered, as well as the type of studies included according to PICOS criteria[22, 23], which is provided in Table 1. The primary objective of this review was the efficacy of AL measured as treatment success at day 28 (or adequate clinical and parasitological response (ACPR). ACPR is defined by the WHO as the “absence of parasitaemia on day 28 irrespective of axillary temperature, in patients who did not previously meet any of the criteria of early treatment failure, late clinical failure or late parasitological failure” [15]. This is also consistent with previous Cochrane Reviews. The secondary endpoints were fever clearance, parasite clearance, and the frequency of adverse drug reactions (ADRs). ADRs were defined as ‘signs and symptoms that first occurred or became more severe after treatment was started’ or ‘as a sign, symptom, or abnormal laboratory value not present on day 0, but which occurred during follow up, or was present on day 0 but became worse during follow up’. Serious adverse events were defined according to International Conference on Harmonization (ICH) guidelines. Studies included in this review are shown in Table 2.

**Table 1:** PICOS strategy and eligibility criteria

PICOS Strategy	Inclusion criteria	Exclusion criteria
P:Population	Participants residing in Ethiopia and having uncomplicated falciparum malaria, irrespective of gender and age group were considered. Microscopy of the peripheral blood smear samples detected mono-infection with a *P. falciparum* parasite count of 1000–100,000/µl	
I: Intervention	Studies using fixed dose compound tablets artemether–lumefantrine (20/120 mg) were included. All participants must have received a standard six-dose regimen of AL over 3 days and were followed up for 28 days	
C:Comparison	Standard treatment, no treatment, not applicable	
O: Outcome	The primary objective of this review was the efficacy of AL measured as treatment success at day 28 [or adequate clinical and parasitological response (ACPR)]. The secondary outcomes were measured based on the parasite clearance time and fever clearance time and the occurrence of adverse events (AEs)	Studies that do not report any treatment success (cure rates) of AL at day-28 as primary outcome
S: Study design	Randomized clinical trials (RCTs), non-randomized single-arm intervention studies (with or without a control group) and prospective cohort studies that reported the therapeutic efficacy of AL for the treatment of uncomplicated falciparum malaria in Ethiopia	All the non-primary literature, retrospective studies, case reports and animal or in vitro experiments were excluded

**Table 2:** Summary characteristics of included studies on the efficacy and safety of artemether-lumefantrine for treatment of uncomplicated *P. falciparum* malaria in Ethiopia from 2004–2020 (N = 1523)

Study [Ref. No]	Study Settings	Study design	Study duration (months)	Inclusion for age	Transmission level	Patient Enrolled (N)^a^	Patient available (n)^b^	Mean Hg	Pf-GMPD	Length of follow up (DAYS)	Super-vision
Abamecha et al. [35]	Ilu-Harar Health Centre, Chewakadistrct, Ethiopia	One arm, prospective study	September-December 2017	Above 6 months of age	Moderate	80	76	11.7	12,374.3	28	Partial
Teklemariam et al. [44]	SetitHumera, Northwest Ethiopia	Single-arm prospective study	October 28, 2014 and January 9, 2015	≥ 6 months of age	High	92	79	13.2	27,798.0	28	Partial
Deressa et al. [42]	Kola Diba Health Centre (KHC) in the Dembia district, Northwest Ethiopia	Prospective cohort study	April 2015 to February 2016	Above 6 months of age	High	80	75	n/a	8377.8	28	Partial
Nega et al. [39]	Metehara Health Centre, Eastern Ethiopia	Open-label single-arm study	October 2014 to January 2015	≥ 6 months of age	Low-moderate	91	85	12.4	11,509.6	28	Partial
Wudneh et al. [49]	Gendewuha (Metema) Health Centre, Northwest Ethiopia	One-arm open-label study	October 2014 to January 2015	Above 6 months of age	Moderate	91	81	13.7	13,441.6	28	Partial
Kancheet al. [48]	Baddessa Health Centre, Wolaita Zone, Southern Ethiopia Wolaita Zone, Southern Ethiopia	One-arm prospective study	February–March 2015	> 5 years old	Moderate	86	88	10.8	4238.8	28	Partial
Mekonnenet al. [38]	Omo Nada health centre in southwestern Ethiopia	Prospective cohort study	August–December 2011	Above 6 months of age	Moderate	88	86	11.6	8404.0	28	Partial
Ebstie et al. 2015 [47]	Bahir Dar district, Northwest Ethiopia	Prospective observational cohort study	March and July 2012	> 5 years old	Moderate	93	89	10.8	8675.3	28	Partial
Getnet et al. [40]	Enfranze Health Centre, Northwest Ethiopia	One-arm, prospective study	January and May 2013	Above 6 months of age	Moderate	134	130	12.3	7898.0	28	Partial
Mulu et al. [45]	Kemisie Health Centre, Northeast Ethiopia	One-arm prospective study	September, 2012 to May, 2013	Above 6 months of age	Moderate	80	80	NR	10,454.0	28	NR
Eshetu et al. [36]	Agaro Health Centre, Jimma Health Centre, Serbo Health Centre, and Asendabo Health Centre	Open-label, single arm study	November 2008 and January 2009 and between August and December 2009	> 1 year	Moderate	348	315	NR	9720.0	28/42	non-supervised
Kinfu et al. [43]	Tumuga health centre Alamata district, Tigrai regional state, North Ethiopia	Prospective cohort study	August–November 2009	Above 6 months of age	Moderate	66	60	N/R	20,672.0	28	Partial
Hwang et al. [37]	Bishoftu Malaria Clinic and Bulbula Health Centre, Oromia Regional State, Ethiopia	Open-label, single arm study	October and November 2009	Above 6 months of age	Moderate	73	71	12.6	16,374.0	28/42	Partial
Assefa et al. [41]	Serbo Health Centre, Kersa District, Southwest, Ethiopia	Prospective cohort study	November 2007 and January 2008	N/R	Moderate	119	112	12.2	22,660.0	28	Partial
Kefyalew et al. [46]	AlabaKulito Health Centre, Southern Ethiopia	Prospective cohort study	October–December 2007	> 1 year	Low -moderate	102	102	11.4	8264.3	28	Partial

N/R, Not reported; TES, therapeutic efficacy study; Hg, Haemoglobin; Pf-GMPD, *Plasmodium falciparum* geometric mean parasite density of asexual parasites per microlitre of blood

^a^*P. falciparum* patients enrolled in study as per manuscript

^b^Patients available for analysis from study

### Data extraction and management

Initial screening of studies was based on the information contained in their titles and abstracts and was conducted by two independent investigators. When the reviewers disagreed, the article was re-evaluated and, if the disagreement persisted, a third reviewer made a final decision. Full-paper screening was conducted by the same independent investigators.

Data were extracted using a case record form (CRF), including four domains: (1) identification of the study (article title; journal title; authors name; country of the study; language, publication year and study setting); (2) methodological characteristics (study design; stated length of follow-up; sample size; gender; age; intervention details; literature quality assessment characteristics; statistical analyses); (3) main findings (treatment success rates; parasite clearance; fever clearance; adverse events) and (4) conclusions. If the outcome data in the original article were unclear, the corresponding author was contacted via email for clarification. A bibliographic software EndNote X5 citation manager (Thomson Reuters, New York, USA) was used to store, organize and manage all the references and ensure a systematic and comprehensive search.

### Methodological quality assessment

Two review authors independently assessed the methodological quality of the selected studies by using methodological quality assessment forms and the criteria outlined in the Cochrane Handbook for Systematic Reviews of Interventions [22, 23].Any disagreements between the two review authors were resolved through discussion. Quality assessment was undertaken using the Newcastle Ottawa Scale (NOS) for observational studies [26] and modified Jadad scale for interventional studies [27].NOS assess the quality under three major headings, namely, selection of the studies (representativeness and the exposure assessment/control selection), comparability (adjustment for main/additional confounders), and outcome/exposure (adequacy of outcome measured, exposure measured vs. self-report) (Additional file 3). The modified Jadad scale included eight items: randomization, blinding, withdrawals, dropouts, inclusion/exclusion criteria, adverse effects and statistical analysis. The reviewers independently assessed the quality of the methodology of included studies (Additional file 4). This study also assessed using Risk of Bias in Non-Randomized Studies of Interventions (ROBINS-I) assessment tool for non-randomized intervention and cohort studies. Studies were ranked as low, moderate, serious, or critical risk of bias in seven domains [28].

### Statistical analysis

OpenMeta Analyst software for Windows [29, 30] was used to perform the meta-analyses. The heterogeneity of the included studies was evaluated using the Cochran Q and I^2^ statistics. The random effects model was used as standard in the determination of heterogeneity between studies [31]. The I^2^ values were expressed in percentages. Heterogeneity was classified as low, moderate and high, with upper limits of 0–25%, 25–50% and > 50% for I^2^, respectively [32, 33]. The method of random effects model was used to combine the included studies.

## Results

### Literature search results

**Fig. 1:**
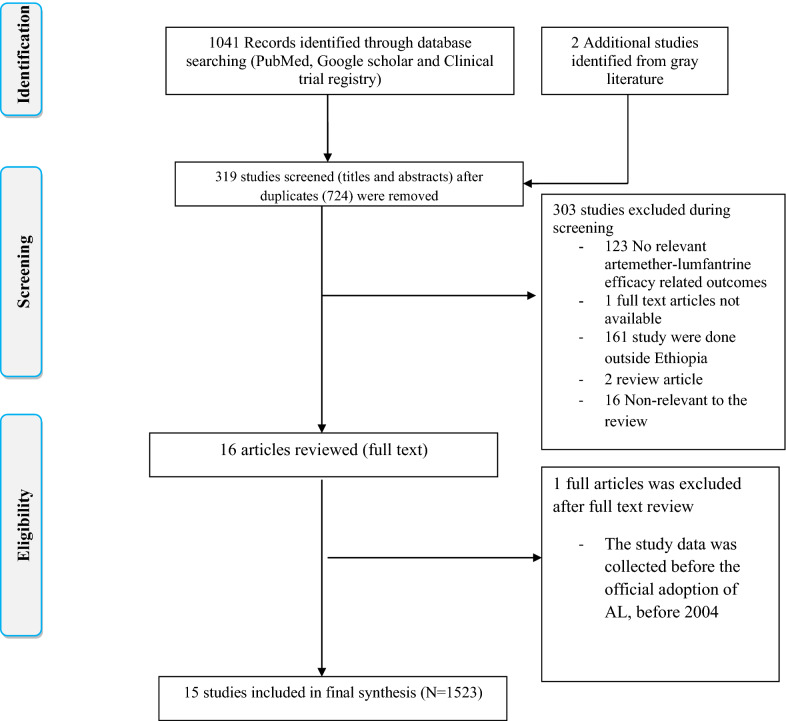
PRISMA flow diagram showing study selection process, 2020

A total of 1043 studies were retrieved from the database and manual searching. Among these, 724 duplicated studies were excluded. From the remaining 319 articles, 303 of them were excluded after evaluation of their title and abstract confirming non relevance to this study. One paper [34] was excluded following full text review as data collection for the study was conducted before official adoption of AL in Ethiopia. Finally, a total of 15 papers met the eligibility criteria and were included in this systematic review and meta-analysis (Fig. 1).

### Characteristics of the included studies

**Fig. 2:**
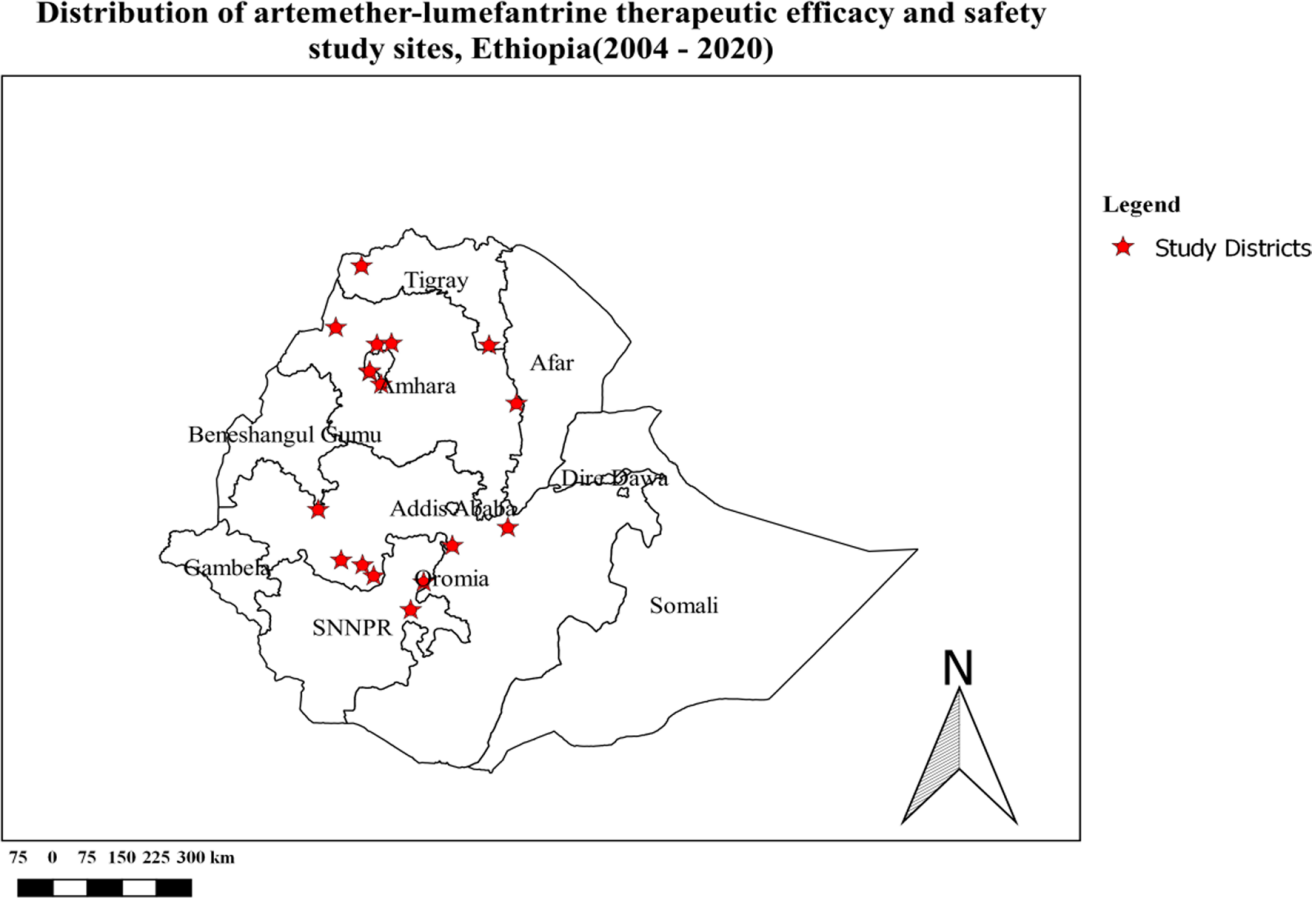
Distribution of artemether-lumefantrine efficacy and safety study sites in Ethiopia from 2004–2020

The summary characteristics of the included studies are shown in Table 2. From 15 eligible studies a total 1523 participants were included. Seven of the studies were interventional [35–41], and the other eight studies were observational study [42–49]. No RCTs had been completed at the time of review. These studies were conducted in different malarious parts of the country with varied transmission intensity (Fig. 2). Most (10/15, 66.7%) of the studies included patients who were ≥ 6 months of age (Table 2). Treatment outcomes in all studies were assessed using clinical and parasitological criteria according to WHO guidelines [15]. In the majority of the studies (86.7%), treatment compliance was assured by supervised administration of the study drug under direct observation on days 1, 2 and 3, i.e. the morning doses were directly observed over 3 days, while the evening doses were given to patients for intake at home by health extension workers. The endpoint was day 28 in all studies [15]. RoB assessment is shown for all studies in Table 5.

### Treatment outcome

**Fig. 3:**
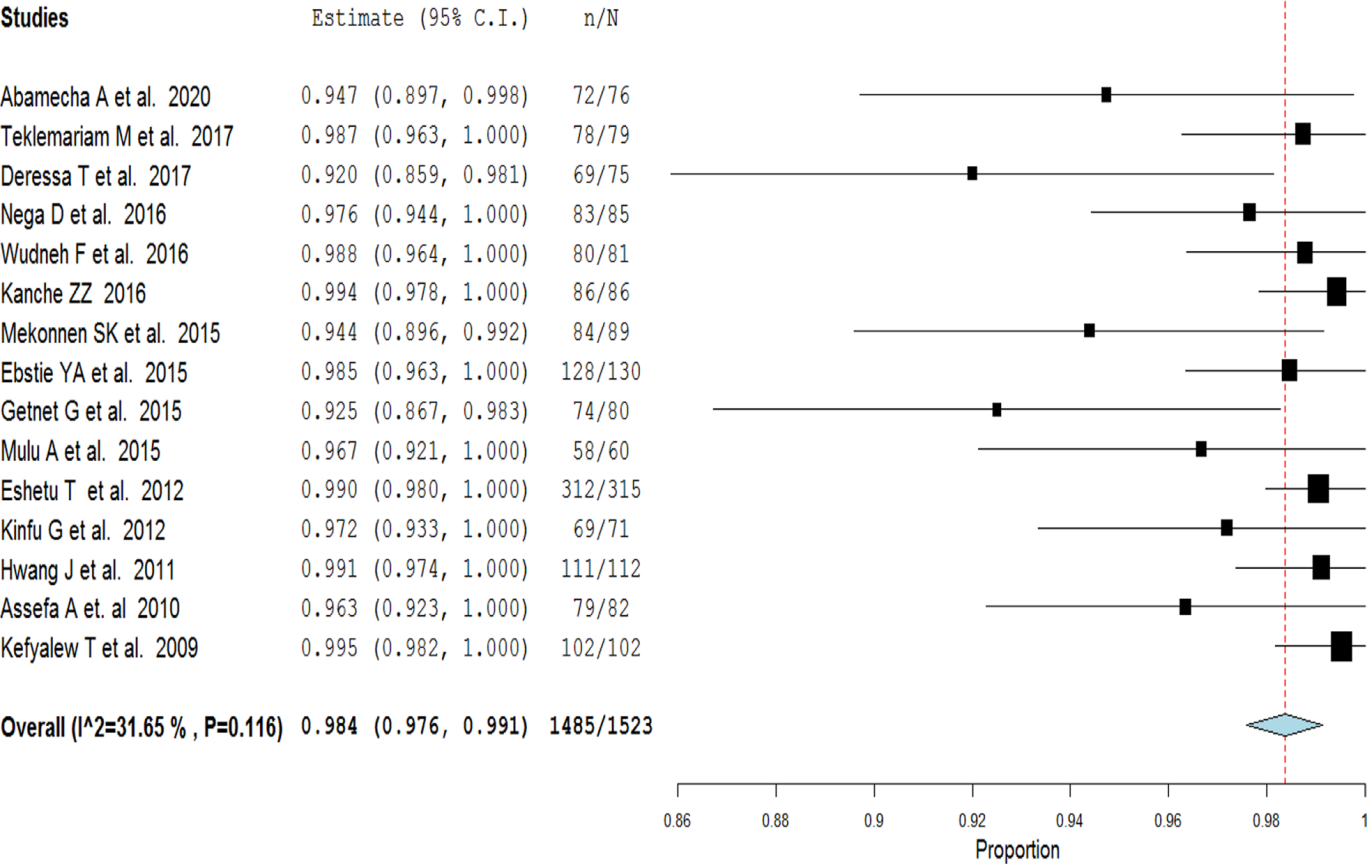
PCR-uncorrected treatment success of artemether-lumefantrine therapy using a random effect model

**Fig. 4:**
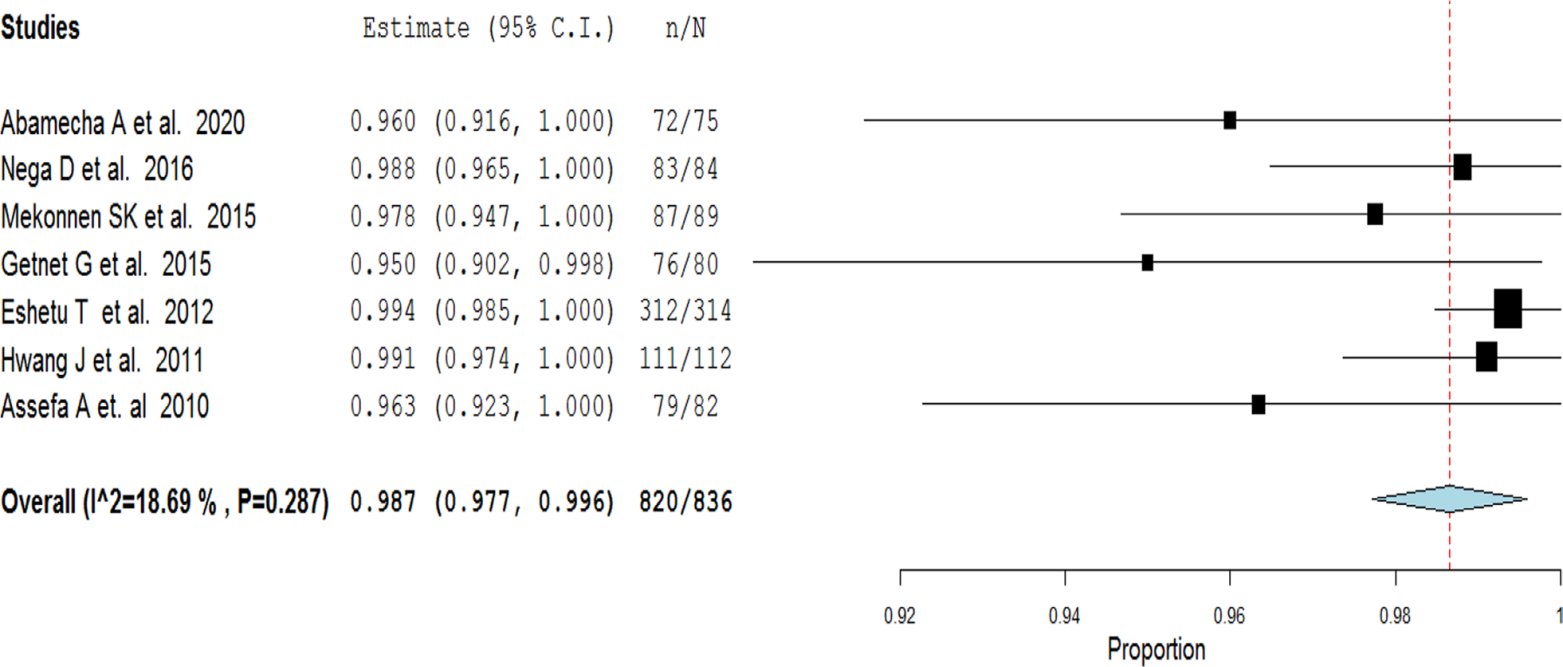
PCR-corrected treatment success of artemether-lumefantrine therapy a random effect model

The overall PCR-uncorrected pooled proportion estimate of treatment success of AL therapy for uncomplicated falciparum malaria was 98.4% (95%CI 97.6–99.1). A random-effects model was used because of substantial heterogeneity [χ^2^ = 20.48, *df* (14), *P* = 0.011 and I^2^ = 31.65; Fig. 3]. PCR-corrected pooled proportion of treatment success of AL therapy was 98.7% (95% CI 97.7–99.6). A random-effects model was used [χ^2^ = 7.37, *df* (6), *P* = 0.287 and I^2^ = 18.69; Fig. 4].

The proportion of recurrence infection was ranging from 1–5.6% at 28-day follow-up period after treatment with AL. The proportion of recurrence infection was ranging from 4.6–6.7% at 42-day follow-up period after treatment with AL.

The PCR-corrected cure rates of AL therapy ranged from 95.0 to 99.4% in per-protocol analysis and 88.8 to 97.4% in intention-to-treat analysis. The percentage of ACPR and the 95% CI are presented in Table 3. The highest cure rate 99.4% (95% CI 97.4–100.0) was reported by study conducted in Jimma Zone, Southwest Ethiopia in 2012 [36], and 97.4% (95% CI 93.9–100) reported by study conducted in Bishoftu Malaria Clinic and Bulbula Health Centre, Oromia Regional State, Ethiopia 2011 [37].

**Table 3:** Treatment Outcome of AL Therapy reported in efficacy studies in Ethiopia

Study	PP PCR-corrected percentage cure rate (95% CI), day-28	ITT PCR-corrected percentage cure rate (95% CI), day-28
Abamecha et al. [35]	96.0(91.6–100)	94.9(90.1–99.8)
Nega et al. [39]	98.8(96.5–100)	92.2(86.7–97.8)
Mekonnen et al. [38]	97.8(94.7–99.8)	96.7(93.0–100)
Getnet et al. [40]	95.0(90.2–100)	97.4(93.9–100)
Eshetu et al. [36]	99.4(97.4–100)	89.9(86.7–93.1)
Hwang et al. [37]	99.1(91.6–100)	94.1(89.9–98.3)
Assefa et al. [41]	96.3(92.3–100)	88.8(82.2–95.3)

### Fever and parasite clearance rate

Among the five partially supervised efficacy studies that reported fever clearance, more than 75% of the patients cleared fever by day 1 post-treatment with AL [38–43]. Some authors did not measure fever clearance on subsequent days post drug administration and only choose day-3 for this clinical measurement [41, 47]. Among the fifteen studies that reported parasite clearance, five studies showed day-3 parasitaemic cases of 5.7%, 5.1%, 5%, 3.9% and 3.8% [35, 40, 42, 47, 48]. Table 4 shows the overall progress of fever and parasite clearance in the first three days of AL treatment.

**Table 4:** Fever and parasite clearance reported in efficacy studies in Ethiopia (2004–2020)

Study	Patient Enrolled (N)	Patient available	Patient Included	Fever clearance (%)			Parasite clearance (%)			Supervised
				D1	D2	D3	D1	D2	D3	
Abamecha et al. [35]	80	76	72	52.5	87.2	97.5	61.2	81.2	96.2	Partial
Teklemariam et al. [44]	92	79	78	80.0	97.8	100.0	33.0	84.4	100.0	Partial
Deressa et al. [42]	80	75	69	62.5	93.7	97.5	67.5	85.0	95.0	Partial
Nega et al. [39]	91	85	83	78.7	94.3	97.7	69.7	95.5	100.0	Partial
Wudneh et al. [49]	91	81	80	69.6	97.8	100.0	23.6	91.0	100.0	Partial
Kanche et al. [48]	88	86	86	N/R	59.1	93.2	N/R	72.2	94.3	Partial
Mekonnen et al*.* [38]	93	89	84	88.1	94.4	100.0	88.8	96.6	100.0	Partial
Ebstie et al. [47]	134	130	128	NR	NR	87.9	NR	85.9	96.1	Partial
Getnet et al. [40]	80	80	74	75.0	91.3	96.2	73.8	91.3	94.9	Partial
Mulu et al. [45]	66	60	58	89.4	98.5	100.0	84.8	93.9	100.0	NR
Eshetu et al. [36]	348	315	312	NR	96.7	99.1	NR	98.2	99.4	Non-supervised
Kinfu et al. [43]	73	71	69	NR	NR	100.0	NR	100.0	100.0	Partial
Hwang et al. [37]	119	112	111	65.2	90.5	93.0	NR	93.1	99.1	Partial
Assefa et al. [41]	90	82	79	NR	NR	100	98	NR	100.0	Partial
Kefyalew et al. [46]	102	102	102	44.1	82.4	93.1	NR	NR	NR	Partial

### Safety outcomes

The current meta-analysis showed that 80% of the included studies reported ADRs to AL which were observed in 36.1%, (550/1523) patients. All of the ADRs were mild and resolved spontaneously. Two SAE were observed (Additional file 5).

### Methodological quality assessment

Eight observational studies [42–49] were assessed with the Newcastle Ottawa Scale (NOS) [26] with satisfactory qualities with a value score of 5 (Additional file 3) and while the remaining seven interventional studies [35–41] were assessed using the modified Jadad scale [27] with high qualities with a value score of 4 (Additional file 4). All or most of the included studies had a ‘serious’ or ‘critical’ risk of bias due to confounding because most were single-arm studies (Table 5).

**Table 5:**
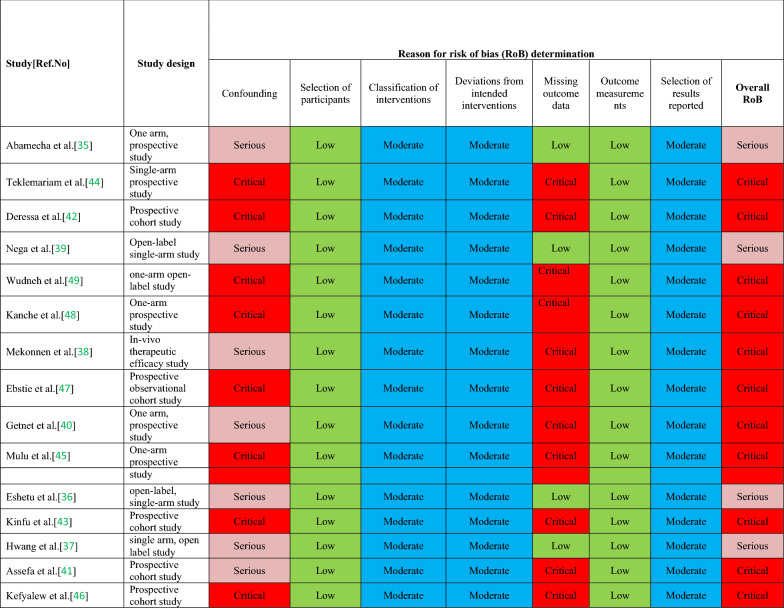
Quality assessment by ‘Risk of bias in non-randomized studies of interventions (ROBIN-I)’ for non-randomized and cohort studies

## Discussion

The present study found high treatment success of AL therapy in the treatment of uncomplicated falciparum malaria in Ethiopia despite its use for more than 16 years. Besides, AL was generally a safe treatment. Previous meta-analysis in 2017 revealed similarly high efficacies of AL [18, 19]. This result is also consistent with neighbouring Sudan, a high treatment success rate (98%) of malaria treatment was recently reported in a meta-analysis that included 20 studies with a total of 4070 patients [50]. The treatment success of 98.7% (95% CI 97.7–99.6) found in this study suggests that, in accordance with WHO parameters [15], AL is still effective as first-line drug for uncomplicated malaria treatment in Ethiopia, but warrants regular monitoring.

There is a concern about the limited post-treatment prophylactic effects of AL in high transmission areas [15]. In this study, the proportion of recurrence infection ranging from 1 to 5.6% at 28-day follow-up period after treatment with AL. From the included studies, two studies [36, 37] also had 42-day follow up period, and the proportion of recurrence infection were relatively high (ranging from 4.6–6.7%)**.** The study results showed that most recurrent parasitaemia occur after day 28 and this emphasizes the need for follow-up periods of at least 42 days. High recurrent parasitaemia rate in children ≤ 5 years (9.4%) was observed, which suggest that the partner drug may not provide prolonged protection despite high therapeutic efficacy [51].This observation has also been reported in Democratic Republic of Congo, which showed high level of resistance to lumefantrine [52]. In most of the studies, a great majority of the recurrent infections were due to re-infections when assessed with a step-wise PCR genotyping protocol. This signifies that the drugs are still efficacious and the high rates of re-infections could only be attributed to high malaria transmission. In terms of clinical practice, the high re-infection rates are of great concern among clinicians. Clinicians should be clearly guided on what to expect and how to handle such cases with recurrent infections within a period of three to eight weeks post-treatment. The observed high re-infection rates after AL treatment underscores the importance of providing anti-malarial drug with a longer period of protection against re-infection, such as DHA-piperaquine [53] and integrating treatment with non-therapeutic prevention and control measures (insecticide-treated bed nets, indoor residual spraying and other vector control measures) to effectively prevent recurrent infections [54, 55]. Besides, it is also important to use transmission-blocking drugs (e.g. use of primaquine) (gametocytocidal) in low transmission areas.

Most studies included in the present review achieved a rapid reduction of fevers and parasitaemia between D0 and D3 of assessment. A previous aggregate study on the clinical predictors of early parasitological response to ACT in African patients with uncomplicated falciparum malaria confirmed the rapid decrease of parasite positivity rate from 59.7% (95% CI 54.5–64.9) on day 1 to 6.7% (95% CI 4.8–8.7) on day 2 and 0.9% (95% CI 0.5–1.2) on day 3 [56].

In resource-limited settings, the day-3 parasite-positive rate can be used as a proxy measure of delayed parasite clearance [57]. In the present review, few studies showed day-3 parasitaemic cases (3.8–5.7%) after treatment with AL [35, 40, 42, 47, 48].However, most of the studies reviewed in this article were based on 24-h sampling, which is not the recommended method for assessing parasite clearance and detection of tolerance/resistance to artemisinins.

Regarding safety of AL for treatment of uncomplicated malaria, mild adverse events (a headache, cough, fever, diarrhoea, vomiting, perioral ulcer, anorexia, abdominal pain, dizziness and nausea, weakness/fatigue and others) were mostly reported in the eligible studies. Besides, almost all were resolved soon after completion of the treatment except cough [35, 41, 44]. Similar mild adverse events have been associated with AL; the most common being headache, fever, vomiting followed by gastrointestinal disturbances [50, 58]. The observed rate of 36.1%, (550/1523) ADRs was comparable with the rate reported in the previous review in Ethiopia where 269 of 633 patients had ADRs, with a pooled event rate of 41.2% [19].

From the included studies, one study reported serious adverse events (SAE) in two infants [36]. These infants had SAE on the day of presentation (day-0) with high parasitaemia (> 95,000/μL), no signs of severe malaria were noticed at admission and did not tolerate oral treatment. After re-dosing and repeated vomiting, the infants were referred to the ward for intravenous treatment; one died the same day. The cause of death was not established and its possible association with AL treatment could not be ascertained.

### Limitation of the review

This review provided an overall country-specific performance of AL after the wide-scale deployment, since 2004 as first-line anti-malarials for treating uncomplicated *P. falciparum* malaria in Ethiopia. The main limitation of this work was the lack of a control group in the included studies that severely limits the ability to draw a firm conclusion regarding the efficacy of an intervention. Moreover, there are insufficient number of therapeutic efficacy studies (TESs) studies with high-quality and more rigorous design. This may be due to the fact that TESs and long-term follow-up of patients require logistics and incur high cost in low and middle income countries, limiting regular implementation of clinical evaluation within the country. The current study however is the first most comprehensive effort at highlighting the levels of implementation of TESs in Ethiopia and provides an overall country-specific performance of AL after their wide-scale deployment since 2004 as first-line anti-malarials for treating uncomplicated *P. falciparum* malaria in the country.

## Conclusions

The present meta-analysis provides some evidence to support that AL therapy is efficacious and safe in treating uncomplicated falciparum malaria in Ethiopia. However, owing to the risk of bias in the included studies, strong conclusions cannot be drawn. Further high-quality randomized controlled trials are warranted to substantiate the efficacy and safety of AL, to detect future changes in parasite sensitivity to AL in Ethiopia.

## Supplementary Information

**Additional file 1.** PRISMA Check list.

**Additional file 2. **Detailed search strategy for the different electronic databases.

**Additional file 3. **Quality assessment of included studies using Newcastle Ottawa Scale (NOS).

**Additional file 4. **Quality assessment of included studies using Modified Jadad Scale.

**Additional file 5. **Safety outcomes of included studies.

## Data Availability

All generated data about the review are included in this manuscript. The original data can be accessed from the corresponding author at any time.

## Abbreviations

ACPR: Adequate clinical and parasitological response
PCR: Polymerase chain reaction
ACT: Artemisinin-based combination therapy
ADRs: Adverse drug reactions
AE: Adverse events
AL: Artemether–lumefantrine
DHP: Dihydroartemisinin–piperaquine
NOS: Newcastle Ottawa Scale
PICOS: Participants/population, Intervention, comparator(s), outcome(s), study design
PRISMA: Preferred reporting items for systematic reviews and meta-analyses
RCT: Randomized controlled trials
ROBINS-I: Risk of Bias in Non-Randomized Studies of Interventions
SAE: Serious adverse events
WHO: World Health Organization

## References

[1] Federal Democratic Republic of Ethiopia Ministry of Health (2016). National malaria elimination roadmap.

[2] Federal Democratic Republic of Ethiopia Ministry of Health (2020). National malaria elimination strategic plan 2021–2025.

[3] WHO Global Malaria Programme (2010). Guidelines for the treatment of malaria.

[4] The WorldWide Antimalarial Resistance Network (WWARN) DP Study Group (2013). The effect of dosing regimens on the antimalarial efficacy of dihydroartemisinin-piperaquine: a pooled analysis of individual patient data. PLoS Med..

[5] White NJ, Olliaro PL (1996). Strategies for the prevention of antimalarial drug resistance: rationale for combination chemotherapy for malaria. Parasitol Today.

[6] White NJ, Nosten F, Looareesuwan S, Watkins WM, Marsh K, Snow RW (1999). Averting a malaria disaster. Lancet.

[7] Federal Democratic Republic of Ethiopia Ministry of Health (2004). Malaria diagnosis and treatment guidelines for health workers in Ethiopia.

[8] Klonis N, Xie SC, McCaw JM, Crespo-Ortiz MP, Zaloumis SG, Simpson JA (2013). Altered temporal response of malaria parasites determines differential sensitivity to artemisinin. Proc Natl Acad Sci USA.

[9] Premji ZG, Kokwaro G, Mwai L, Nzila A, Efferth T, White N (2009). Coartem®: the journey to the clinic. Malar J.

[10] Bridgford JL, Xie SC, Cobbold SA, Pasaje CFA, Herrmann S, Yang T (2018). Artemisinin kills malaria parasites by damaging proteins and inhibiting the proteasome. Nat Commun.

[11] Golenser J, Waknine JH, Krugliak M, Hunt NH, Grau GE (2006). Current perspectives on the mechanism of action of artemisinins. Int J Parasitol.

[12] Shandilya A, Chacko S, Jayaram B, Ghosh I (2013). A plausible mechanism for the antimalarial activity of artemisinin: a computational approach. Sci Rep.

[13] White NJ (1994). Clinical pharmacokinetics and pharmacodynamics of artemisinin and derivatives. Trans R Soc Trop Med Hyg.

[14] Eckstein-Ludwig U, Webb RJ, Van Goethem IDA, East JM, Lee AG, Kimura M (2003). Artemisinins target the SERCA of *Plasmodium falciparum*. Nature.

[15] WHO (2009). Methods for surveillance of antimalarial drug efficacy.

[16] Federal Democratic Republic of Ethiopia Ministry of Health (2012). National malaria guideline.

[17] President’s Malaria Initiative Ethiopia (2020). Malaria Operational Plan FY.

[18] Ayalew MB (2017). Therapeutic efficacy of artemether-lumefantrine in the treatment of uncomplicated *Plasmodium falciparum* malaria in Ethiopia: a systematic review and meta-analysis. Infect Dis Poverty.

[19] Gebreyohannes EA, Bhagavathula AS, Seid MA, Tegegn HG (2017). Anti-malarial treatment outcomes in Ethiopia: a systematic review and meta-analysis. Malar J.

[20] Moher D, Liberati A, Tetzlaff J, Altman DG, PRISMA Group (2009). Preferred reporting items for systematic reviews and meta-analyses: the PRISMA Statement. BMJ.

[21] Abamecha A, Yilma A, Addisu W, Yewhalaw D, Abdissa A. Monitoring of efficacy and safety of artemether-lumefantrine for treatment of uncomplicated *P. falciparum* malaria in Ethiopia: a systematic review and meta-analysis of the evidence. PROSPERO 2020 CRD42020201859.https://www.crd.york.ac.uk/prospero/display_record.php?ID=CRD42020201859. Accessed 3 Aug 2020

[22] Higgins JPT, Thomas J, Chandler J, Cumpston M, Li T, Page MJ, Welch VA (editors). Cochrane handbook for systematic reviews of interventions version 6.0 (updated July 2019). Cochrane; 2019. www.training.cochrane.org/handbook. Accessed 3 Mar 2020

[23] The Cochrane Collaboration. Cochrane handbook for systematic reviews of interventions version 5.1.0 (2011); 2011. http://handbook.cochrane.org/. Accessed 3 Mar 2020

[24] Federal Ministry of Health of Ethiopia (2004). Malaria diagnosis and treatment guidelines for health workers.

[25] Lefebvre C, Manheimer E, Glanville J. Searching for studies. In: Higgins JPT, Greene S, Cochrane handbook for systematic reviews of interventions, Version 5.0. eds; 2008.

[26] Wells GA, Shea B, O’Connell D, Peterson J, Welch V, Losos M, Tugwell P. The Newcastle-Ottawa Scale (NOS) for assessing the quality if nonrandomized studies in meta-analyses; 2012. http://www.ohrica/programs/clinical_epidemiology/oxfordasp. Accessed 2 Dec 2020

[27] Jadad AR, Moore RA, Carroll D, Jenkinson C, Reynolds DJ, Gavaghan DJ (1996). Assessing the quality of reports of randomized clinical trials: is blinding necessary?. Control Clin Trials.

[28] Sterne JA, Hernán MA, Reeves BC, Savović J, Berkman ND, Viswanathan M (2016). ROBINS-I: a tool for assessing risk of bias in non-randomised studies of interventions. BMJ.

[29] Open-Meta-Analyst. http://www.cebm.brown.edu/openmeta/doc/openMA_help.html#self. Accessed 3 Mar 2020

[30] Wallace BC, Schmid CH, Lau J, Trikalinos TA (2009). Meta-Analyst: software for meta-analysis of binary, continuous and diagnostic data. BMC Med Res Methodol.

[31] Ryan R. Cochrane Consumers and Communication Review Group. Heterogeneity and subgroup analyses in Cochrane Consumers and Communication Group reviews: planning the analysis at protocol stage; 2016. http://cccrg.cochrane.org. Accessed 12 Feb 2019

[32] Kontopantelis E, Springate DA, Reeves D (2013). A re-analysis of the Cochrane Library Data: the dangers of unobserved heterogeneity in meta-analyses. PLoS ONE.

[33] Higgins JPT, Thompson SG (2002). Quantifying heterogeneity in a meta-analysis. Stat Med.

[34] Jima D, Tesfaye G, Medhin A, Kebede A, Argaw D, Babaniyi O (2005). Safety and efficacy of artemether-lumefantrine in the treatment of uncomplicated falciparum malaria in Ethiopia. East Afr Med J.

[35] Abamecha A, Yilma D, Addisu W, El-Abid H, Ibenthal A, Noedl H (2020). Therapeutic efficacy of artemether-lumefantrine in the treatment of uncomplicated *Plasmodium falciparum* malaria in Chewaka District, Ethiopia. Malar J.

[36] Eshetu T, Abdo N, Bedru KH, Fekadu S, Wieser A, Pritsch M (2012). Open-label trial with artemether-lumefantrine against uncomplicated *Plasmodium falciparum* malaria three years after its broad introduction in Jimma Zone, Ethiopia. Malar J.

[37] Hwang J, Alemayehu BH, Hoos D, Melaku Z, Tekleyohannes SG, Teshi T (2011). In vivo efficacy of artemether-lumefantrine against uncomplicated *Plasmodium falciparum* malaria in Central Ethiopia. Malar J.

[38] Mekonnen SK, Medhin G, Berhe N, Clouse RM, Aseffa A (2015). Efficacy of artemether-lumefantrine therapy for the treatment of uncomplicated *Plasmodium falciparum* malaria in Southwestern Ethiopia. Malar J.

[39] Nega D, Assefa A, Mohamed H, Solomon H, Woyessa A, Assefa Y (2016). Therapeutic efficacy of artemether-lumefantrine (Coartem®) in treating uncomplicated P falciparum malaria in Metehara, Eastern Ethiopia: regulatory clinical study. PLoS ONE.

[40] Getnet G, Fola AA, Alemu A, Getie S, Fuehrer HP, Noedl H (2015). Therapeutic efficacy of artemether-lumefantrine for the treatment of uncomplicated *Plasmodium falciparum* malaria in Enfranze, north-west Ethiopia. Malar J.

[41] Assefa A, Kassa M, Tadese G, Mohamed H, Animut A, Mengesha T (2010). Therapeutic efficacy of artemether/lumefantrine (Coartem^®^) against *Plasmodium falciparum* in Kersa, South West Ethiopia. Parasit Vectors.

[42] Deressa T, Seid ME, Birhan W, Aleka Y, Tebeje BM (2017). In vivo efficacy of artemether–lumefantrine against uncomplicated *Plasmodium falciparum* malaria in Dembia District, northwest Ethiopia. Ther Clin Risk Manag.

[43] Kinfu G, Gebre-selassie S, Fikrie N (2012). Therapeutic efficacy of artemether-lumefantrine for the treatment of uncomplicated *Plasmodium falciparum* malaria in Northern Ethiopia. Malar Res Treat.

[44] Teklemariam M, Assefa A, Kassa M, Mohammed H, Mamo H (2017). Therapeutic efficacy of artemether-lumefantrine against uncomplicated *Plasmodium falciparum* malaria in a high-transmission area in northwest Ethiopia. PLoS ONE.

[45] Mulu A, Geresu B, Beyene Y, Ademe M (2015). Efficacy of artemether-lumefantrine for the treatment of uncomplicated *Plasmodium falciparum* malaria in Northeast Ethiopia. Int J Basic Clin Pharmacol.

[46] Kefyalew T, Animut A, Tamene T, Jima D, Hailemariam A, Legesse M (2009). Efficacy of six-dose regimen of artemether-lumefantrine for the treatment of uncomplicated falciparum malaria, three years after its introduction into Ethiopia. Parasite.

[47] Ebstie YA, Zeynudin A, Belachew T, Desalegn Z, Suleman S (2015). Assessment of therapeutic efficacy and safety of artemether-lumefantrine (Coartem^®^) in the treatment of uncomplicated *Plasmodium falciparum* malaria patients in Bahir Dar district, Northwest Ethiopia: an observational cohort study. Malar J.

[48] Kanche ZZ, Woticha EW, Gidebo KD (2016). Therapeutic efficacy and safety of artemether–lumefantrine (Coartem®) in uncomplicated *Plasmodium falciparum* malaria in Wolaita Zone, Southern Ethiopia. J Biol Agric Healthc.

[49] Wudneh F, Assefa A, Nega D, Mohammed H, Solomon H, Kebede T (2016). Open-label trial on efficacy of artemether/lumefantrine against the uncomplicated *Plasmodium falciparum* malaria in Metema district, Northwestern Ethiopia. Ther Clin Risk Manag.

[50] Adam I, Ibrahim Y, Gasim GI (2018). Efficacy and safety of artemisinin-based combination therapy for uncomplicated *Plasmodium falciparum* malaria in Sudan: a systematic review and meta-analysis. Malar J.

[51] Ngasala BE, Malmberg M, Carlsson AM, Ferreira PE, Petzold MG, Blessborn D (2011). Efficacy and effectiveness of artemether-lumefantrine after initial and repeated treatment in children < 5 years of age with acute uncomplicated *Plasmodium falciparum*malaria in rural Tanzania: a randomized trial. Clin Infect Dis.

[52] Plucinski MM, Talundzic E, Morton L, Dimbu PR, Macaia AP, Fortes F (2015). Efficacy of artemether-lumefantrine and dihydroartemisinin-piperaquine for the treatment of uncomplicated malaria in children in Zaire and Uíge Provinces, Angola. Antimicrob Agents Chemother.

[53] Okell LC, Cairns M, Griffin JT, Ferguson NM, Tarning J, Jagoe G (2014). Contrasting benefits of different artemisinin combination therapies as first-line malaria treatments using model-based cost-effectiveness analysis. Nat Commun.

[54] WHO (2018). World malaria report.

[55] WHO (2015). Policy Brief on single-dose primaquine as a gametocytocide in *Plasmodium falciparum* malaria.

[56] Dahal P, Dalessandro U, Dorsey G, Guerin PJ, Nsanzabana C, WWARN Artemisinin based Combination Therapy (ACT) Africa Baseline Study Group (2015). Clinical determinants of early parasitological response to ACTs in African patients with uncomplicated falciparum malaria: a literature review and meta-analysis of individual patient data. BMC Med.

[57] WHO (2011). Global plan for artemisinin resistance containment (GPARC).

[58] Shayo A, Buza J, Ishengoma DS (2015). Monitoring of efficacy and safety of artemisinin-based anti-malarials for treatment of uncomplicated malaria: a review of evidence of implementation of anti-malarial therapeutic efficacy trials in Tanzania. Malar J.

