# Supplementary material for: Efficacy and safety of artemether–lumefantrine for treatment of uncomplicated Plasmodium falciparum malaria in Ethiopia: a systematic review and meta-analysis
Source: Malar J. 2021 May 6;20:213. doi: 10.1186/s12936-021-03745-8 (PMC8101141; doi:10.1186/s12936-021-03745-8)
Supplement: Supplementary file 2 — Additional file 2. Detailed search strategy for the different electronic databases. [file 12936_2021_3745_MOESM2_ESM.docx]

Additional file 2: Detailed search strategy for the different electronic databases

| ***No.*** | ***Databases (Total 3)*** | ***Search Terms*** | ***Search results***  ***Total = 1041*** | ***Date of search*** |
| --- | --- | --- | --- | --- |
| **1** | **PubMed** | (((((((Efficacy[MeSH Terms]) OR (Therapeutic efficacy[MeSH Terms])) AND (Artemether-lumefantrine[MeSH Terms])) OR (Coartem[MeSH Terms])) AND (Plasmodium falciparum malaria[MeSH Terms])) OR (falciparum malaria[MeSH Terms])) AND (Antimalarial drug[MeSH Terms])) AND (Ethiopia) | **44** | **15/09/2020** |
| **2** | **Google Scholar** | 1. **With all of the words:** ("Therapeutic efficacy" AND "Artemether-lumefantrine" AND "Ethiopia") 2. **With at least one of the words:** “"Plasmodium falciparum" AND “falciparum malaria” | **545 + 451 = 996** | **15/09/2020** |
| **3** | **Clinical Trial.gov** | Condition or disease: Plasmodium falciparum, Ethiopia  Other terms: artemether-lumefantrine | 1 | 15/09/2020 |
